# The osteoclastic activity in apical distal region of molar mesial roots affects orthodontic tooth movement and root resorption in rats

**DOI:** 10.1038/s41368-024-00284-1

**Published:** 2024-02-28

**Authors:** Wenhao Zheng, Xiaofeng Lu, Guangjin Chen, Yufeng Shen, Xiaofei Huang, Jinfeng Peng, Jiajia Wang, Ying Yin, Wencheng Song, Mengru Xie, Shaoling Yu, Lili Chen

**Affiliations:** 1 Department of Stomatology, Union Hospital, Tongji Medical College, Huazhong University of Science and Technology, Wuhan, China; 2 School of Stomatology, Tongji Medical College, Huazhong University of Science and Technology, Wuhan, China; 3 Hubei Province Key Laboratory of Oral and Maxillofacial Development and Regeneration, Wuhan, China; 4 Department of Stomatology, The First Affiliated Hospital, School of Medicine, Shihezi University, Shihezi, China

**Keywords:** Bone, Bone quality and biomechanics

## Abstract

The utilization of optimal orthodontic force is crucial to prevent undesirable side effects and ensure efficient tooth movement during orthodontic treatment. However, the sensitivity of existing detection techniques is not sufficient, and the criteria for evaluating optimal force have not been yet established. Here, by employing 3D finite element analysis methodology, we found that the apical distal region (A-D region) of mesial roots is particularly sensitive to orthodontic force in rats. Tartrate-resistant acidic phosphatase (TRAP)-positive osteoclasts began accumulating in the A-D region under the force of 40 grams (g), leading to alveolar bone resorption and tooth movement. When the force reached 80 g, TRAP-positive osteoclasts started appearing on the root surface in the A-D region. Additionally, micro-computed tomography revealed a significant root resorption at 80 g. Notably, the A-D region was identified as a major contributor to whole root resorption. It was determined that 40 g is the minimum effective force for tooth movement with minimal side effects according to the analysis of tooth movement, inclination, and hyalinization. These findings suggest that the A-D region with its changes on the root surface is an important consideration and sensitive indicator when evaluating orthodontic forces for a rat model. Collectively, our investigations into this region would aid in offering valuable implications for preventing and minimizing root resorption during patients’ orthodontic treatment.

## Introduction

Nowadays, there is a growing demand for orthodontic treatment, especially among appearance-conscious adults. Thus, this brings greater challenges for orthodontists as adults have lower tissue responsiveness to orthodontic forces compared to adolescents and this difficulty arises from the need to balance therapeutic effects with potential side effects.^1–3^ Orthodontic tooth movement (OTM) depends on the periodontal ligament (PDL), which is a local aseptic inflammatory associated bone remodeling process.^4^ Too little orthodontic force may fail to produce an effective biological response from PDL, while excessive force can lead to adverse reactions such as hyalinization, root resorption, and anchorage loss.^5–8^ Hence, an optimal orthodontic force (OOF) for OTM has been proposed as the lightest force that still provides a maximum or a near-maximum biological response.^8^ However, evidences have not reached a consensus regarding the exact magnitude of the OOF and it is urgent to explore how to evaluate it in clinical practice.^9,10^

OTM occurs when the PDL senses mechanical stimuli and initiates a coordinated alveolar bone adaptation, involving osteoclastic activity on the compressive side and osteogenesis on the tensive side.^11–13^ Alveolar bone resorption on the compressive side is considered to be the physiological basis of OTM.^13^ It has been shown that the biological response of PDL relying on mechanical state regulates OTM efficiency.^14^ Therefore, it is essential to clarify the stress distribution and biological response of periodontal tissue under orthodontic forces to define the OOF. Previous studies on the OOF have mainly been conducted by animal experiments.^11,15,16^ However, the stress distribution of PDL responding to forces is complicated at the histological level due to the irregular anatomy of the tooth-periodontium system.^17,18^ Thus, it is hard to distinguish the region of tension or compression by traditional analysis methods. Recently, a more effective method of numerical simulation by 3D finite element analysis (FEA) has been applied to directly reveal the stress distribution of PDL.^19^ In a rat model, the compressive region was found to be distributed throughout the entire mesial root (MR) apex and the cervical margin of its mesial side.^14^ FEA has been proven to be an adaptable, accurate, flexible, and less time-consuming tool for dental biomechanical study. However, a more detailed regional division of the PDL and the associated biological response are still unclear and warrant further investigation.

In the present study, we combined FEA with deep micro-computed tomography (micro-CT) analysis to develop a new method of biomechanical analysis. This study aims to investigate the biological activities in compressive regions of roots and alveolar bone in response to orthodontic force and their relationship with force magnitudes. Using multimodal analysis, we have identified the specific regions for focused investigation and proposed a new strategy for predicting personalized OOF.

## Results

### The pressure zone distributes in the cervical mesial and apical distal regions of mesial roots during orthodontic tooth movement

**Fig. 1:**
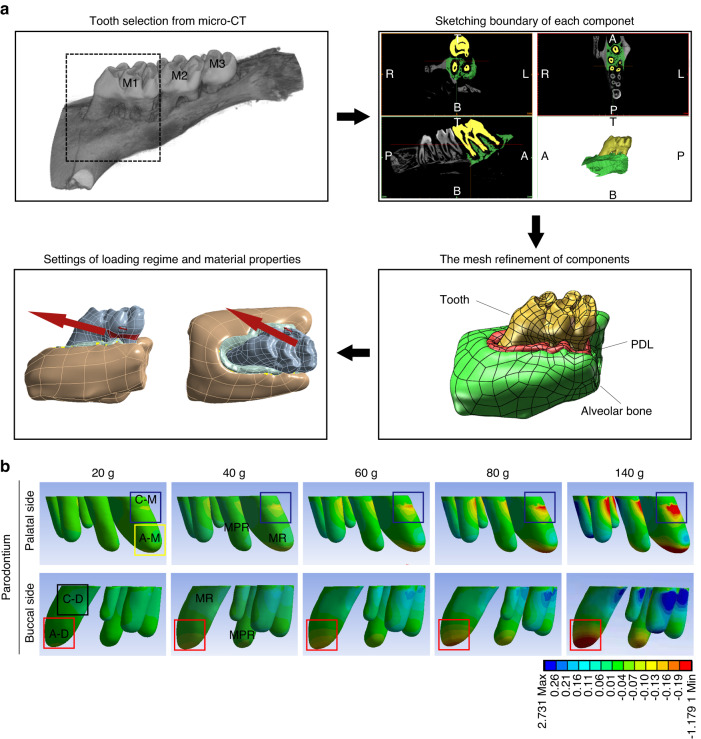
Compressive stress is distributed in the C-M and the A-D regions of mesial roots for a rat OTM model. **a** Illustration of the model creation process from micro-computed tomography images. The TIFF images are imported into MIMICS software and the maxillary M1 is selected as the object of study. Thresholding and mask editing are used to separate the tooth and the alveolar bone. The periodontal ligament model is established by Boolean operation. All components are imported into ANSYS software to refine the mesh and set the loading regime (red arrows). PDL periodontal ligament, M1 the first molar, M2 the second molar, M3 the third molar, A anterior, P posterior, T top, B bottom, L left, R right. **b** Finite element analysis of periodontal ligament with different force magnitudes. Compressive stress in the A-D region is shown in the red box and the C-M region is shown in the blue box. The yellow box represents the A-M region and the black box represents the C-D region. MR the mesial root, MPR the mesial palatal root, A-M apical mesial region, C-M cervical mesial region, A-D apical distal region, C-D cervical distal region

In early studies, it has been considered that the pressure zone is near the mesial side while the tension zone is near the distal side in a rat orthodontic model.^13^ To investigate the stress distribution in the PDL of multirooted teeth which is responsible for alveolar bone remodeling, as well as facilitating tooth movement, the first molar (M1) from micro-CT was selected and FEA was conducted (Fig. 1a). We were surprised to find that the compressive stress in the PDL was not only distributed in the cervical mesial region (C-M region) of all roots but also in the apical distal region (A-D region) of the MR and the mesial palatal root (MPR) (Fig. 1b), which was different from the previous literature.^13^ Besides, the tensile stress was distributed in the apical mesial region (A-M region) and the cervical distal region (C-D region) of MR and MPR. Interestingly, the rest roots showed the compressive stress limited to the C-M region. The stress distribution of both MR and MPR different from the other roots indicated that there might be more complicated alveolar bone resorption and molar movement.

### Orthodontic force promotes molar tipping by alveolar bone resorption around the A-D region of mesial roots

**Fig. 2:**
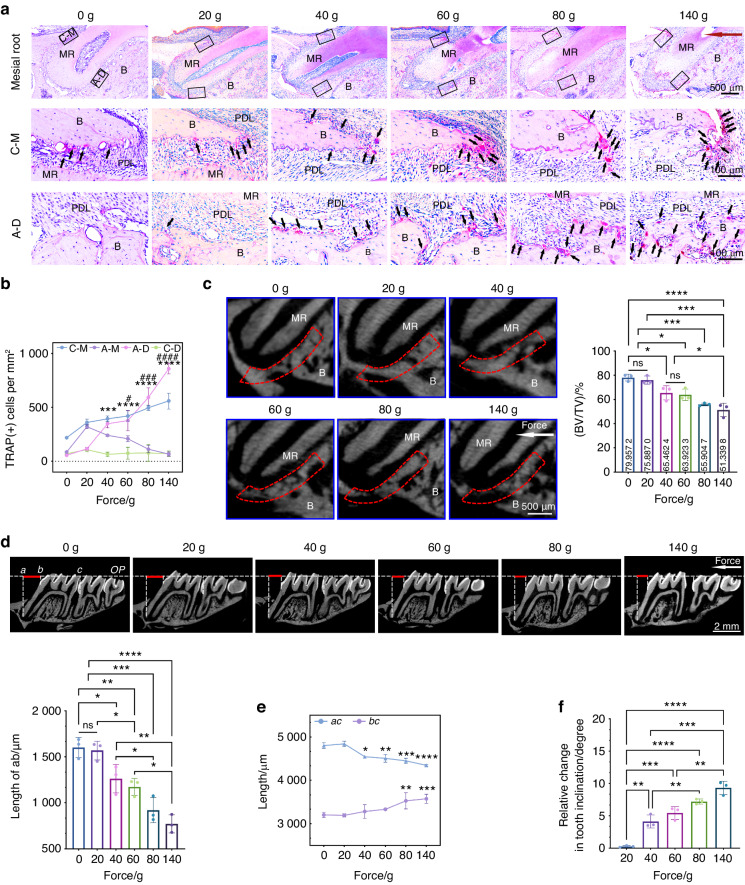
The osteoclastic activity in the A-D region of MR on the alveolar bone surface affects orthodontic tooth tipping. **a** TRAP staining images show the distribution of osteoclasts (black arrows) on the surface of alveolar bone varies along with force magnitudes and the most obvious change is reflected in the A-D region. The red arrow represents the orthodontic force orientation. C-M cervical mesial region, A-D apical distal region, MR mesial root, B alveolar bone, PDL periodontal ligament. **b** Statistics of TRAP-positive cells on the alveolar bone surface in regions of C-M, A-M, A-D and C-D. A-M apical mesial region, C-D cervical distal region. Mean ± SEM, *n* = 3. * Represents the statistics in the A-D region and ^#^ represents the C-M region. All the statistics come from the comparison with the group of 0 g. ***P < 0.001, ****P < 0.000 1, ^#^*P* < 0.05, ^###^*P* < 0.001, ^####^*P* < 0.000 1 by two-way ANOVA. **c** Micro–computed tomography images show the level of alveolar bone resorption in the A-D region. Statistics of bone morphology-related parameter BV/TV is shown. BV bone volume, TV total volume, mean ± SD, *n* = 3. ns, not significant, *P < 0.05, ***P < 0.001, ****P < 0.000 1 by one-way ANOVA. **d** Taking the mesial cementoenamel junction of the second molar as a reference, *ac* represents the displacement of the mesial root apex, and *bc* represents the displacement of the crown. The relative displacements between the crown and the apex (the red lines represent *ab*) are shown in micro-CT images. OP occlusal plane. The statistics show the length of *ab* among groups, mean ± SD, *n* = 3. ns, not significant, *P < 0.05, **P < 0.01, ***P < 0.001, ****P < 0.000 1 by one-way ANOVA. **e** Statistics of length of *ac* and *bc* indicate the reverse displacement of the root apex increases from 40 g. All the statistics come from the comparison with the group of 0 g. Mean ± SD, *n* = 3. *P < 0.05, **P < 0.01, ***P < 0.001, ****P < 0.000 1 by two-way ANOVA. **f** Statistics of relative tooth inclination after 2-week force loading, mean ± SD, *n* = 3. **P < 0.01, ***P < 0.001, ****P < 0.000 1 by one-way ANOVA

To confirm whether bone resorption happens around both the A-D region and the C-M region of MR, we conducted a histological analysis of osteoclastogenesis in rats. An accumulation of tartrate-resistant acidic phosphatase (TRAP) positive cells in the A-D region and the C-M region was observed through tissue section staining (Fig. 2a, S1a). At a force of 20 grams (g), there were almost no osteoclasts appearing in the A-D region, indicating that the compressive stress in this region was insufficient to induce osteoclastic activity. TRAP-positive cells were observed in the C-M region in every group. However, the number of osteoclasts in the A-D region increased more significantly than that in the C-M region as the force increased (Fig. 2a, b). Accordingly, the number of osteoclasts in the A-D region was more strongly positively correlated with the force value than that in the C-M region (Fig. S1b). Meanwhile, it was consistent with the micro-CT results, showing significant alveolar bone resorption in the A-D region (Fig. 2c). We extended the duration to four weeks to determine whether loading duration could change the effect of force on osteoclasts and alveolar bone resorption. The number of TRAP-positive cells on the bone surface in the A-D region still increased significantly from 40 g (Fig. S1c, S1d), which was similar to our previous results. The percentage of TRAP-positive cells in the A-D region was highest, suggesting that bone resorption occurred mainly in this region (Fig. S1e). Similarly, the number of osteoclasts in the A-D region was highly positively correlated with force magnitudes (Fig. S1f). With the increase of force value, alveolar bone resorption in the A-D region increased more significantly with 4-week loading than that with 2-week loading (Figs. 2c, S1g, S1h).

To explore the relationship between inflammation and TRAP-positive osteoclasts, we observed when the heavier force (140 g) was applied, pro-inflammatory M1-like macrophages (CD86^+^) were remarkably infiltrated in the A-D region of periodontal tissues, while there was no significant change in the group applied with lighter force (40 g) (Fig. S2a). In contrast, anti-inflammatory M2-like macrophages (CD206^+^) were slightly infiltrated in the A-D region of 40 g group, while there was no change in the 140 g group (Fig. S2b). Additionally, immunohistochemistry (IHC) showed that the expression of pro-inflammatory factors IL-1β, TNF-α, and IL-6 in the A-D region of periodontal tissues increased obviously in the 140 g group, while there was no difference in the 40 g group. Taken together, the periodontal tissues in the A-D region of the heavier force group presented a more severe inflammatory tendency (Fig. S2c–e). To better understand how the condition of the PDL varies under different forces, which can be exploited to optimize tooth movement, we investigated the expression of IL-1β, TNF-α, and IL-6 in the C-M region of PDL by IHC staining. Interestingly, there was no statistical difference in the expression of IL-1β among groups, which has been proved to be the key indicator of periodontitis.^20–22^ High expression level of TNF-α in the group of 140 g was shown, while no significant difference existed among the groups in the results of IL-6 (Fig. S3a–c). This also explained that excessive compressive stress in the C-M region would not lead to more inflammation. These results indicated that the TRAP-positive osteoclasts were more sensitive to force magnitudes in the A-D region than that in the C-M region.

Previous studies have verified that the type of OTM in rats is tipping.^23^ We measured the relative displacement between root and crown and the results showed a sharp decrease from 40 g (Fig. 2d). Interestingly, the cervix moved along the direction of the loading force while the apex moved in the opposite way (Fig. 2e). The measurement of tooth inclination was showed (Figs. S4a, 4b). We found that the 40 g group showed a rapid increase in the relative inclination compared with the 20 g group and the relative inclination became greater as the force increased (Fig. 2f), which was consistent with the TRAP staining and the alveolar bone parameter data. These results indicated that alveolar bone resorption in A-D region affected molar movement and would be a clear indicator of OTM.

### The root surface in the A-D region is most sensitive to force

**Fig. 3:**
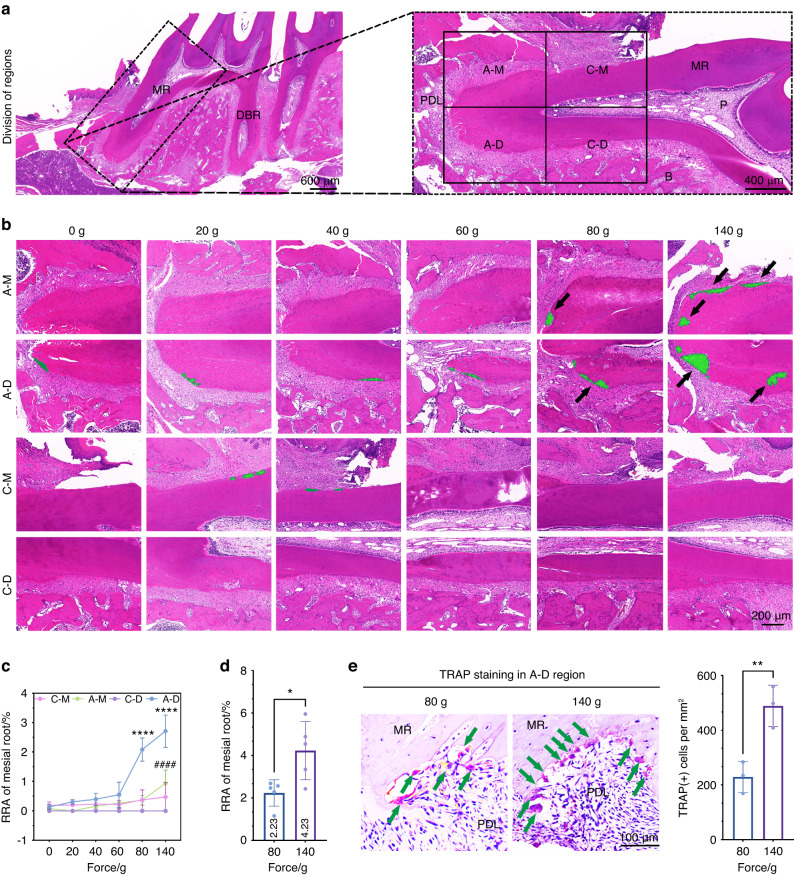
The changes on the root surface of A-D region are most sensitive to force magnitudes. **a** Schematic diagram of region division is shown. PDL periodontal ligament, DBR distal buccal root, MR mesial root, P pulp, B alveolar bone. A-M apical mesial region, C-M cervical mesial region, A-D apical distal region, C-D cervical distal region. **b** HE staining images of four regions in which the area of root resorption covered by green (black arrows) is mainly distributed in the A-D region after 2-week force loading. **c** Statistics of root resorption area of A-M, C-M, A-D, and C-D regions in MR. RRA root resorption area. * Represents the statistics of RRA in the A-D region, ^#^ represents the statistics of RRA in the A-M region and comes from the comparison with the group of 0 g. All the statistics come from the comparison with the group of 0 g, mean ± SD, *n* = 4–5. ****P < 0.000 1, ^####^*P* < 0.000 1 by two-way ANOVA. **d** Statistics of RRA of the entire mesial root, mean ± SD, *n* = 5. *P < 0.05 by Student’s *t*-test. **e** TRAP staining images of both 80 g group and 140 g group show the osteoclasts on the root surface (green arrows) in the A-D region. The data of the A-D region is calculated, mean ± SD. *n* = 3. **P < 0.01 by Student’s *t*-test

Considering the significant changes in compressive stress of PDL around the MR apex with different loadings, we speculated that MR resorption might also be involved in the A-D region. To confirm this hypothesis, we conducted histological morphological analysis (Fig. 3a) and found obvious root resorption with forces ≥80 g. It was the A-D region where MR resorption occurred most. The resorption in the A-M region happened in the group of 140 g (Fig. 3b, c). Besides, the group of 140 g showed a larger root resorption area than that in the 80 g group (Fig. 3d). Obviously, TRAP-positive cells accumulated on the root surface in the A-D region when the force reached 80 and 140 g (Figs. 3e, S5a). It was observed that the root resorption area and TRAP-positive cell accumulation increased more significantly after 4-week force loading (Fig. S5b, S5c). All these findings suggested the changes on the root surface in the A-D region were more sensitive to force than other regions.

### Root resorption mainly occurs in the A-D region

**Fig. 4:**
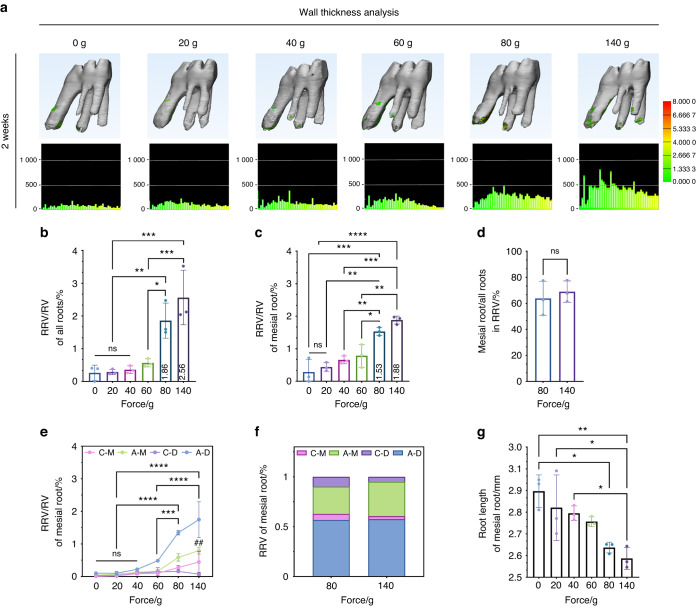
The vast majority of root resorption induced by excessive orthodontic force occurs in the A-D region. **a** The wall thickness analysis of root resorption of the first molar after 2-week force loading. **b** Statistics of root resorption volume percentages of all roots after two-week force loading. RRV root resorption volume, RV root volume, mean ± SD, *n* = 3. ns, not significant, *P < 0.05, **P < 0.01, ***P < 0.001 by one-way ANOVA. **c** Statistics of root resorption volume percentages of mesial roots, mean ± SD, *n* = 3. ns, not significant, *P < 0.05, **P < 0.01, ***P < 0.001, ****P < 0.000 1 by one-way ANOVA. **d** The root resorption volume proportion of the mesial root comparing to all roots, mean ± SD, *n* = 3. ns, not significant by Student’s *t*-test. **e** Statistics of root resorption volume of mesial roots in different regions. * represents the statistics in the A-D region. ^#^ Represents the statistics in the A-M region and comes from the comparison with the group of 0 g, mean ± SEM, *n* = 3. ns, not significant, ***P < 0.001, ****P < 0.000 1, ^##^ *P* < 0.01 by two-way ANOVA. **f** The root resorption volume percentages of four regions in the mesial root under 80 and 140 g. The root resorption proportions in the A-D region are higher than others. *n* = 3. **g** Statistics of root length of mesial roots, mean ± SD, *n* = 3. *P < 0.05, **P < 0.01 by one-way ANOVA

To accurately assess root resorption, 3D modeling and wall thickness analysis were carried out to investigate the volume changes of root resorption. The results showed root resorption occurred with excessive forces (≥80 g) (Fig. 4a–c). There was almost no root resorption in the groups of 0, 20, 40, and 60 g. The level of total root resorption reached from 1.86% to 3.20% at 80 g and 2.56% to 3.91% at 140 g, respectively after 2- or 4-week treatment (Figs. 4b, S5d, S5e). Among the five roots, root resorption in MR accounted for the majority of the total root resorption (Figs. 4c, 4d, S5f). In addition to the most obvious increase of MR resorption in the A-D region, the A-M region also surprisingly showed an increase under the force of 140 g, which indicated that the resorption around the entire MR apex had occurred (Fig. 4e). Similarly, the majority of root resorption occurred in the A-D region (Fig. 4f). On the other hand, the length of MR started to shorten from 80 g, also indicating root resorption. (Fig. 4g). After 4-week force loading, more severe root resorption was observed in A-D region and A-M region, resulting in a more decrease in the MR length (Fig. S5g, S5h). These results suggested that the A-D region was the main region where root resorption occurs and prolonging force duration enhanced the effect of force magnitudes on root resorption.

### The force of 40 g can be optimal in orthodontic molar movement of rats

**Fig. 5:**
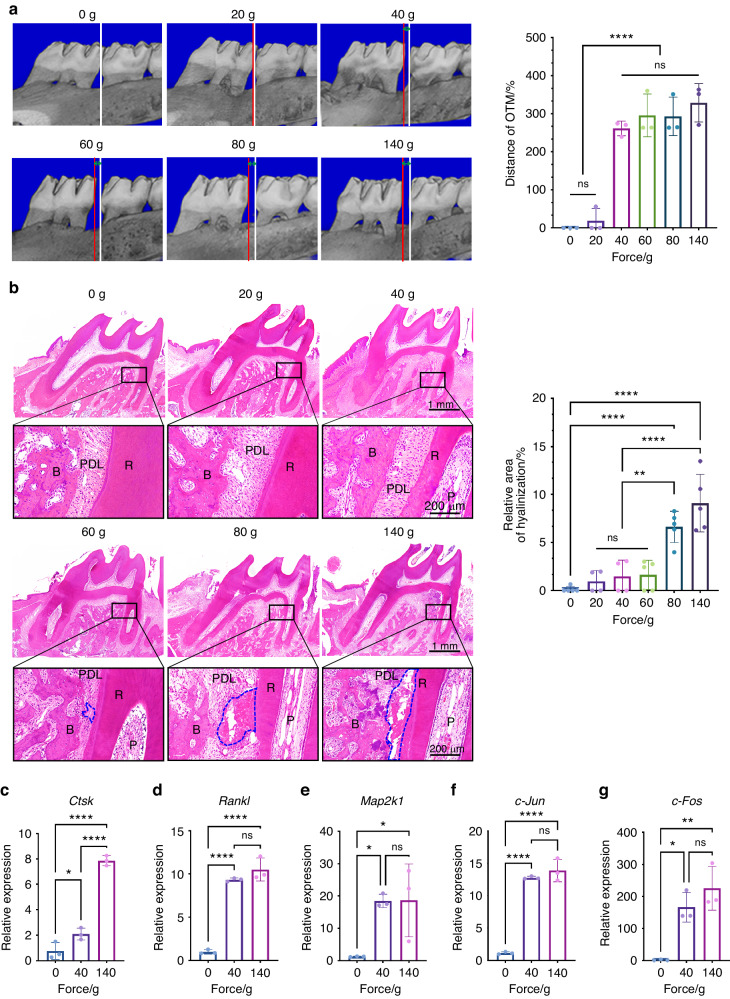
The optimal orthodontic force is 40 g in a rat model. **a** Micro-computed tomography images after 2-week force loading. The distance between the white line and the red line represents the distance of orthodontic tooth movement. The statistical results show no difference among the groups of 40 g and above. OTM orthodontic tooth movement. Mean ± SD, *n* = 3. ns, not significant, ****P < 0.000 1 by one-way ANOVA. **b** The area covered by a blue dot line represents hyalinization. R root, B bone, P dental pulp, PDL periodontal ligament. Mean ± SD, *n* = 4–5. ns, not significant, **P < 0.01, ****P < 0.000 1 by one-way ANOVA. **c–g** Gene relative expression results of *Ctsk*, *Rankl*, *Map2k1*, *c-Jun and c-Fos* in the alveolar bone around the mesial roots indicate that the mRNA expression levels by mechanical force are upregulated. Mean ± SD, *n* = 3. ns, not significant, *P < 0.05, **P < 0.01, ****P < 0.000 1 by one-way ANOVA

Our previous results have confirmed that significant alveolar bone resorption in the A-D region happened with no root resorption under the forces ranged 40–60 g, indicating that orthodontic force affected OTM and root resorption mainly through the biological response of the A-D region. We speculated that the force of 40–60 g might be the OOF and the A-D region could be used as an observation area to evaluate it. To test our conjecture, micro-CT analysis was combined with side effect analysis. Micro‐CT data showed a sharp increase in the OTM distance from the force of 40 g after 2-week loading, with no significant difference among the groups treated with 40 g or higher forces (Fig. 5a). This suggested that increasing orthodontic forces did not facilitate more tooth displacement. In addition, hematoxylin–eosin (HE) staining showed that the hyalinization area increased in the groups of 80 and 140 g force (Fig. 5b). Consistently, we noticed that the expression of bone resorption indicators in the A-D region, including cathepsin K (*Ctsk*) and receptor activator for nuclear factor-kappa B ligand (*Rankl*), was up-regulated with force loading (Fig. 5c, d). It was noticed that there was no substantial difference between 40 and 140 g in *Rankl* expression (Fig. 5d). Moreover, the collagen synthesis and cellular proliferation in the A-D region increased most under the force of 40 g (Fig. S6a, S6b).

The mitogen-activated protein kinase (MAPK) family is heavily involved in various cellular activities, such as cell proliferation, differentiation, and motility. It also participates in the regulation of mechanotransduction.^24,25^ Given this, we speculated that MAPK may play an important role in affecting osteoclast activity in the A-D region. Quantitative real-time polymerase chain reaction (qRT-PCR) analysis was performed to examine the expression of mitogen-activated protein kinase kinase 1 (*Map2k1*), c-*Jun* and *c-Fos* under different forces. Compared with the 0 g, we observed a significant increase at the forces of 40 and 140 g, but there was no significant difference between 40 and 140 g (Fig. 5e–g). Molecules involved in mechanical signal transduction (*Map2k1*, c-*Jun*, and *c-Fos*) showed the same change as *Rankl* (Fig. 5d–g). This may explain why heavy force could not facilitate more OTM than light force. In conclusion, our findings verified that 40 g could be the most effective force for OTM in rats. As the region of compressive stress concentration, the A-D region could be a promising focus point to access tooth movement or predict personalized OOF.

## Discussion

Variables such as force magnitude, direction, distribution, duration, initial tooth displacement, stress and biologic changes of the PDL can play roles in OTM.^10^ Although a number of factors have been reported to affect the efficiency of OTM, force is the most controllable factor in clinical practice.^26^ In this study, we investigated the effects of various force magnitudes and different durations on the structural characteristics of alveolar bone, the stress distribution in the PDL, the morphological changes of roots and OTM pattern. We found that the osteoclastic activity in the A-D region of MR regulates OTM and root resorption under various forces. The force of 40 g is the minimum to cause effective tooth movement with less root resorption and hyalinization, which can be considered as the OOF. Taken together, our results proposed osteoclastic activity in the A-D region as a promising indicator for predicting the OOF.

OTM is a synergistic result of physical phenomenon and biological responses of the tooth–alveolus complex by externally applied forces.^27^ Osteoclastic activities and bone resorption on the compressive side are considered to be the limitation that determines the velocity of OTM. The positive correlation between compressive stress and osteoclast activities in PDL has previously been demonstrated at the cellular level, but few studies have been carried out in animals.^18^ Our results verified that force magnitudes affect not only the number of osteoclasts but also the distribution. Almost no alveolar bone resorption in the A-D region at the group of 20 g, resulting in no distal movement of the apex. Nevertheless, when the force reached 40 g or greater, the apex of MR started moving in the opposite direction of force and the molar began moving and tipping. The A-D region of the MR became the main compressive region and the apex became the fulcrum of the whole tooth movement.

According to the Burstone formula (M:F = 0.068 × *h*^2^/*D*),^13,28^ the height of the alveolar bone and the distance from the center of resistance to the center of rotation impact the patterns of OTM crucially. Notably, excessive forces (≥80 g) caused not only alveolar bone resorption in the A-D region but also root resorption in the entire apical region, resulting in a shorter root length. As an inherent property, the center of resistance is affected by the force system and histologic morphologies of roots and supporting tissues.^29,30^ Magnitudes and directions of orthodontic forces bring about changes in the center of resistance and then the center of rotation, inducing different patterns of tooth movement.^31^ Consistent with previous studies, the molar OTM in rats is tipping.^11,23,32^ Additionally, we specified that the molar tipping is characterized by the MR apex moving in the reverse direction. This suggests that excessive forces (≥80 g) may result in changes in the center of resistance and the center of rotation, and thus aggravate molar inclination by affecting both alveolar bone resorption in the A-D region and root length. In addition to the magnitude and direction of orthodontic force, the unique morphology of the M1 may contribute to the important role that the A-D region plays in OTM. Although the morphology of human teeth is different from that of rat molars, the innovative approach in this study deserves to be applied to analyze every human tooth. In future studies, we can conduct finite element analysis and histological analysis on teeth with different shapes in large animals (such as beagles) to find key areas that play similar roles as the A-D region in this study. On this basis, the more complex finite element analysis of human teeth could be carried out. During the orthodontic treatment, the root resorption and bone destruction in the key areas could be monitored by X-ray to direct the individual adjustment.

The evidence is insufficient regarding an OOF range over the years. For human canines, the forces of 75 and 150 g are considered to be within the optimal range, and there was no significant change in the biochemical indicators of bone turnover after force application.^9,33,34^ Some literature studied the OOF for human molars.^35^ In these papers, scholars believed that forces of 50–250 cN for patients showed similar OTM rates. Considering the movement rate, patients’ comfort, and reduction of side effects, 50–100 cN might be optimal.^8^ It was proved in FEA that there was a certain degree of similarity in stress distribution of rat and human molars although the root morphology differs.^36^ However, the main force-bearing roots of human molars during tipping movement require further analysis. Some literatures believe that the force of >50 g is overloaded for rat molars and a force of <25 g is recommended.^37,38^ Alikhani et al. showed a significant increase in the distance between the first and the second right maxillary molars at 10 cN.^15^ In other studies that used rat models, the magnitude of orthodontic force usually ranged from 10 to 100 g.^15,16,39–42^ Meanwhile, given the measurement accuracy of the digital dynamometer, we chose these different forces to conduct this study (0, 20, 40, 60, 80, 140 g). Surprisingly, we found that almost no tooth movement at 20 g while the significant movement started from 40 g with no root resorption and hyalinization in rat models. In this study, the orthodontic appliances were checked daily and reactivated weekly, while these were checked daily without reactivation and animals with loose springs were excluded in Alikhani et al. Moreover, the distal drift of molars may counteract the effects of too-light loading force.^16,43,44^ In addition, Gudhimella et al. induced tooth steady movement with 3 cN by inserting a 1.2 mm × 4 mm self-threading Stryker titanium screw into the alveolar bone.^45^ These are likely to be the reasons for the indicated discrepancy. As suggested, the ways of force loading, duration, accuracy of measuring device and the specification of springs were closely related to the effects of OTM.^10^ Hyalinization, which could be found throughout the whole process of OTM, has always been studied.^46–48^ Our research revealed that hyalinization could also occur within the light force groups (<80 g). We speculate that a small amount of hyalinization may be a concomitant phenomenon of OTM in rats, while a large number will lead to the stagnation of tooth movement. Together, these results indicated that 40 g is likely to be the OOF in a rat model.

The duration of force application is an important factor in optimizing OTM with less root resorption.^7^ We observed that root resorption in the A-D region is located in the cellular cementum layer at 2-week loading, while dentin is involved at 4-week loading, resulting in irreversible root resorption. This is consistent with previous research that continuous forces lead to more root resorption,^49–51^ which reminds us to minimize the duration of treatment in clinical practice. Our study revealed the role of biological response in the A-D region and may provide a new indicator for predicting the pattern of OTM and evaluating personalized OOF.

## Materials and methods

### Animal experimental design

Male, 6-week-old Sprague-Dawley rats were purchased from Beijing Vital River Laboratory Animal Technology Company. The rats were randomly divided into A–F groups with 6 rats in each group. Weigh the weight first and then number it according to the weight from light to heavy. Then use the random function in EXCEL to group randomly. Rats were maintained under specific pathogen-free (SPF) conditions with standard rodent chow and provided free access to water in 12-h light and dark cycles. All animal experimental protocols complied with the guidelines for animal experiments (IACUC number: 2639).

After 2 weeks of adaptation to the environment, the rats were anesthetized by intraperitoneal injection of pentobarbital sodium (40 mg/kg body weight) and orthodontic appliances were installed as previously described.^25^ Considering the continuous eruption of rodents’ incisors will bring a high drop-out rate of the orthodontic appliances, additional undercuts and ligations were performed at the gingival margin of incisors. The activated force value of the coil spring was measured with a digital dynamometer as accurately as possible. In order to diminish the impact caused by mesial displacement of the tooth, the orthodontic appliances were checked daily and reactivated weekly. After 2-week loading duration, the maxillary specimens were collected for the following experiments. The same protocols were performed again for long-term experiments (4-week loading duration).

### Micro-computed tomography

The maxillary specimens were harvested and fixed in 4% paraformaldehyde for 24 h and immersed in 75% ethyl alcohol for micro-CT scanning (SkyScan 1176, Bruker, Germany). The scans were performed at 70 kV, 350 μA, 180° rotation, 0.3° rotation step, and a resolution of 9 μm. After reconstructing the original image datasets by NRecon (Bruker, Belgium), DataViewer (Bruker, Belgium) and CTAn (Bruker, Belgium) were utilized to calculate OTM-related parameters, root resorption volume and create 3D models for the following analysis. Each result was measured three times.

### Finite element analysis

The original files from micro-CT were imported into MIMICS 20.0 software (Materialise, Leuven, Belgium). The teeth and alveolar bone were distinguished preliminarily by setting the gray value thresholding. Models of the M1 and alveolar bone were established in STL format by manipulating region growth, manual selection of tissue boundary, and 3D reconstruction. The models were imported into GeomagicStudio13 software for the NURBS surfaces and finally transformed into a CAD model in STEP format after smoothing and noise reduction. Then the 3D models were constructed in the ANSYS workbench software, which consisted of 83,530 homogenous, isotropic tetrahedral-node solid elements and 139,145 nodes (Table 1). The material properties of the tooth, bone, and PDL were taken referring to the relevant paper.^52^ Stress distribution analyses were performed in this software.

**Table 1:** Element and node number

Model	Node	Element
Tooth	44 100	26 724
PDL	51 243	30 110
Alveolar bone	43 802	26 696
Total	139 145	83 530

### Histological and immunohistochemical evaluation

The maxillary samples of rats fixed in 4% paraformaldehyde were decalcified in 10% neutral ethylene diamine tetraacetic acid (EDTA) solution for 6 weeks. Then the samples were paraffin-embedded, sectioned, and stained in the following experiments. Sample sections were sequentially deparaffinized in xylene, dehydrated in ethanol, and incubated in citrate buffer for 10 min at 90 °C. HE staining was performed for histological and histomorphometric analysis.

TRAP staining was performed to mark osteoclasts using the TRAP kit following the manufacturer’s instructions (Solarbio). Briefly, the TRAP incubation solution was prepared by mixing AS-BI buffer, GBC solution, and TRAP buffer in a 10:1:90 ratio. After dewaxed and rehydrated, the sections were rinsed with TRAP incubation solution at 37 °C for 45–60 min and washed with ultrapure water. Sections were then re-dyed with hematoxylin solution for 5 min. After sealing the slides were imaged immediately.

The sections were incubated with the primary antibody of CD86 (1:400, CY5238, Abways), CD206 (1:100, 60143-1-Ig, Proteintech), IL-6 (1:200, GB11117, Servicebio), IL-1β (1:500, GB11113, Servicebio), TNF-α (1:200, bs-10802R, Bioss), COL-I (1:200, ab270993, Abcam), and Ki67 (1:200, ab16667, Abcam). Then horseradish peroxidase (HRP)-coupled secondary antibodies were used to detect the corresponding primary antibodies. Finally, the samples were visualized by the chromogenic substrate diaminobenzidine (DAB) substrate.

The images were captured using a slide scanner and quantity analyses were conducted using the Image J software. The MR of M1 is divided into four regions: the cervical mesial region (C-M region), the apical mesial region (A-M region), the apical distal region (A-D region), and the cervical distal region (C-D region).

### Measurement of OTM

OTM distance was defined as the distance between the nearest contact points of the M1 and the homolateral second molar (M2) referring to other relevant studies.^11,37,45^

### Measurement of tooth inclination-related parameters

Determination of the measurement layer of relative displacement and occlusal plane (OP) was conducted by DataViewer (Bruker, Belgium). The red line is determined by the MR of M1 and the mesial buccal root (MBR) of the M2. The intersections of perpendiculars from the apex of the MR, the mesial cementoenamel junctions (CEJs) of M1 and M2 to OP were named point *a*, point *b*, and point *c*, representing locations of the root apex, the crown, and the reference position, respectively. The distance of *ab* was defined as the relative displacement between the crown of M1 and the apex of MR, revealing the degree of tooth inclination to a certain extent (Fig. S4a).

Determination of the measurement layer of inclination of M1 was conducted by DataViewer (Bruker, Belgium). The red line is determined by the MPR of the M1 and the M2. The inclination of M1 was determined by the long axes of M1 and M2 on the *X*–*Y* plane. The long axis of M1 was drawn from the midpoint of two CEJs to the apical foramen of the MPR. The long axis of M2 was marked as the long axis of MPR of M2. The angular changes between the experimental groups and 0 g groups were identified as the relative inclination of M1^45^ (Fig. S4b).

### Morphometric parameters analysis of alveolar bone

The binarized datasets on the *X*–*Y* plane were imported into CTAn analysis software. The region of interest (ROI) of the A-D region, including 50 layers, centering on the median plane of the MR, is bounded by the bone surface, the half-length of the root, and a thickness of 400 μm from the PDL.^53^ The parameter (bone volume/total volume, BV/TV) was calculated in 3D analysis.

### Root resorption analysis

The calculation method of the root resorption area in tissue slices was similar to that in previous papers.^11,54^ To further accurately quantify root resorption volume in three dimensions, we extracted the reconstruction datasets of roots and ran convex hull algorithm scripts in MATLAB R2017a (MathWorks, Natick, MA) to generate a new one, which repaired craters on root surface. The root volume (RV) and the root resorption volume (RRV) were calculated and the 3D models of RRV were created by CTAn software. The wall thickness analysis was conducted by importing the model in 3-Matic analysis software (Materialise, Plymouth, MI, USA). The RRV of MR in different regions relied on the ROI drawn manually. In addition, considering the limitation of the convex hull algorithm which could not calculate images in every direction but only the cross-section, the root length in the sagittal section was taken into account and thus the RRV in total was comprehensively evaluated.

### Quantitative real-time polymerase chain reaction (qRT-PCR)

The fresh maxillary specimens were maintained at −80 °C. Total RNA was extracted from alveolar bone tissue around M1 by the TRIzol method. Complementary DNA (cDNA) was acquired by reverse transcription of RNA via the HiScript III RT SuperMix (R323-01, Vazyme), and then SYBR Green detection reagent (Q711-02, Vazyme) was applied for qRT-PCR analysis. The relative expression levels were calculated using the 2^−ΔΔCt^ method. *Gapdh* expression was used for the normalization of results. Table 2 shows the primer sequences.

**Table 2:** The primers used for qRT-PCR

Primer	Forward (5′–3′)	Reverse (5′–3′)
*Gapdh*	ACAGCAACAGGGTGGTGGAC	TTTGAGGGTGCAGCGAACTT
*Ctsk*	GGAGACATGACCAGCGAA	GCCCAACAGGAACCACAC
*Rankl*	TCGGAGGAGATGGGCAGT	GAACATGAAGCGGGAGGC
*Map2k1*	AGGATGATGACTTTGAGAA	CTATGTACGGGGAGTTGCA
*c-Jun*	ACGCCAACCTCAGCAACTT	GTCTGCGGCTCTTCCTTCA
*c-Fos*	TCAAGAACATTAGCAACA	CATAGAAGGAACCAGACA

### Statistical analysis

All results were presented as mean and standard deviation or standard error of the mean. The results were applied to the Shapiro–Wilk test for normal distribution and the Brown–Forsythe test for homogeneity of variance before Student’s *t*-test and one-way or two-way analysis of variance (ANOVA). *P* < 0.05 was set as the threshold to indicate significant differences among groups. All statistical analyses were performed using GraphPad Prism software version 9.1.0 (GraphPad Inc.).

### Supplementary information

Supplementary Figures and Figure legends
