# Supplementary material for: The osteoclastic activity in apical distal region of molar mesial roots affects orthodontic tooth movement and root resorption in rats
Source: Int J Oral Sci. 2024 Feb 28;16:19. doi: 10.1038/s41368-024-00284-1 (PMC10901898; doi:10.1038/s41368-024-00284-1)
Supplement: Supplementary file 1 — Supplementary Figures and Figure legends [file 41368_2024_284_MOESM1_ESM.docx]

**
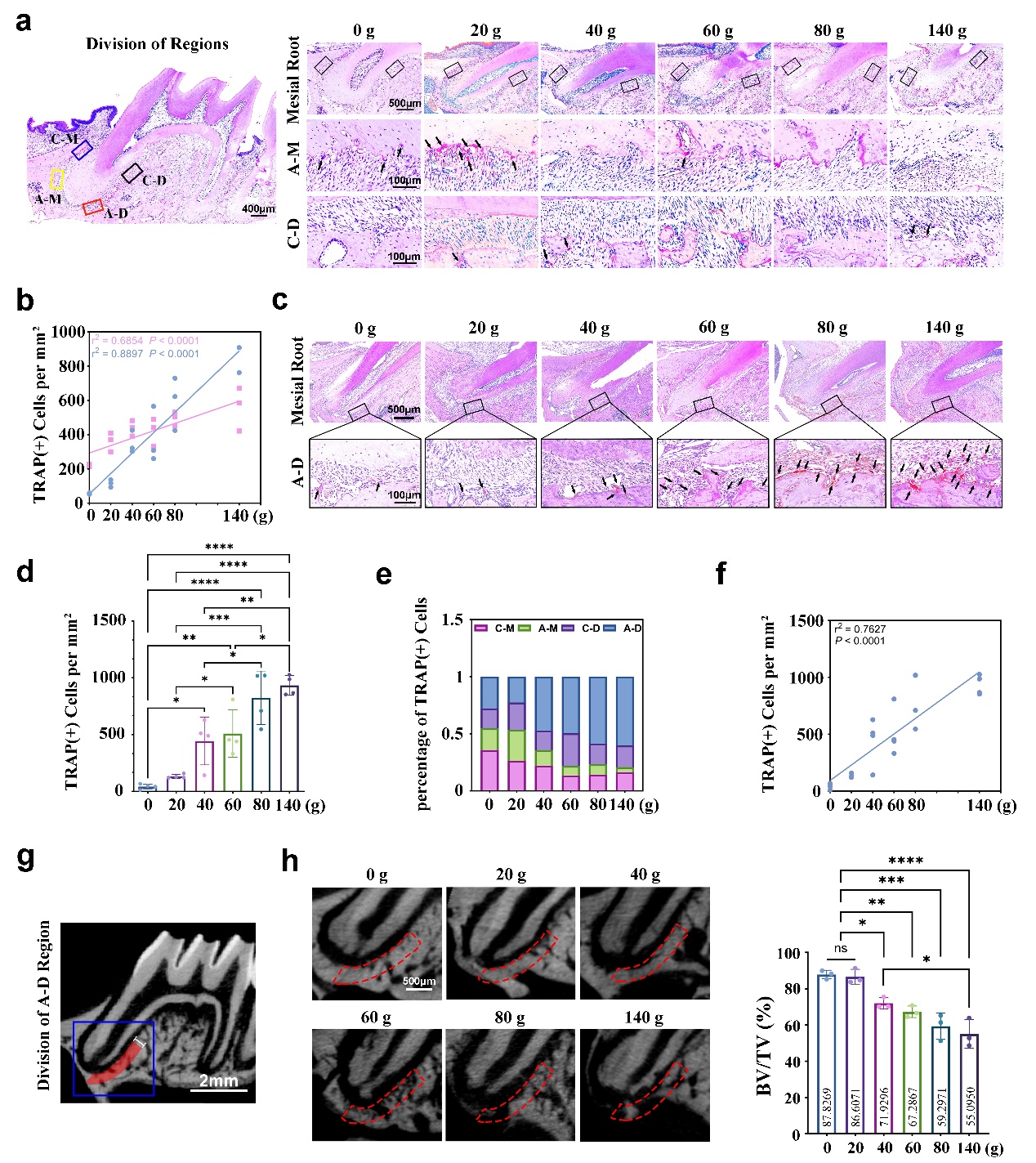
Supplementary Fig. 1 The osteoclastic activity and alveolar bone resorption in the A-D region of MR is enhanced by prolonged force duration.**

1. The left image shows division of regions around mesial roots. TRAP staining images after two-week force loading show osteoclasts in the A-M and the C-D regions. A-M apical mesial region, C-D cervical distal region.
2. Correlation analysis of the TRAP positive cells number per mm^2^ and force magnitudes after two-week force loading. The results show stronger positive correlation exists in the A-D region. The blue line represents the A-D region and the pink line represents the C-M region. A-D apical distal region, C-M cervical mesial region.
3. TRAP staining images show positive cells in the A-D region on the bone surface (black arrows) after four-week force loading.
4. Statistics of TRAP positive cells in the A-D region on the bone surface. Mean ± SD. n=4. **P*<0.05, ***P*<0.01, ****P*<0.001, *****P*<0.0001 by one-way ANOVA.
5. The percentages of TRAP positive cells in different regions after four-week force loading. n=4.
6. Correlation analysis of the TRAP positive cells number per mm^2^ and force magnitudes in the A-D region after four weeks.
7. Division method of region of interest labeled by red area, representing the A-D region.
8. Micro–computed tomography images show the alveolar bone resorption in the A-D region after four weeks. Statistics of bone morphology-related parameter BV/TV. BV bone volume, TV total volume. Mean ± SD. n=3. ns, not significant, **P*<0.05, ***P*<0.01, ****P*<0.001, *****P*<0.0001 by one-way ANOVA.

**
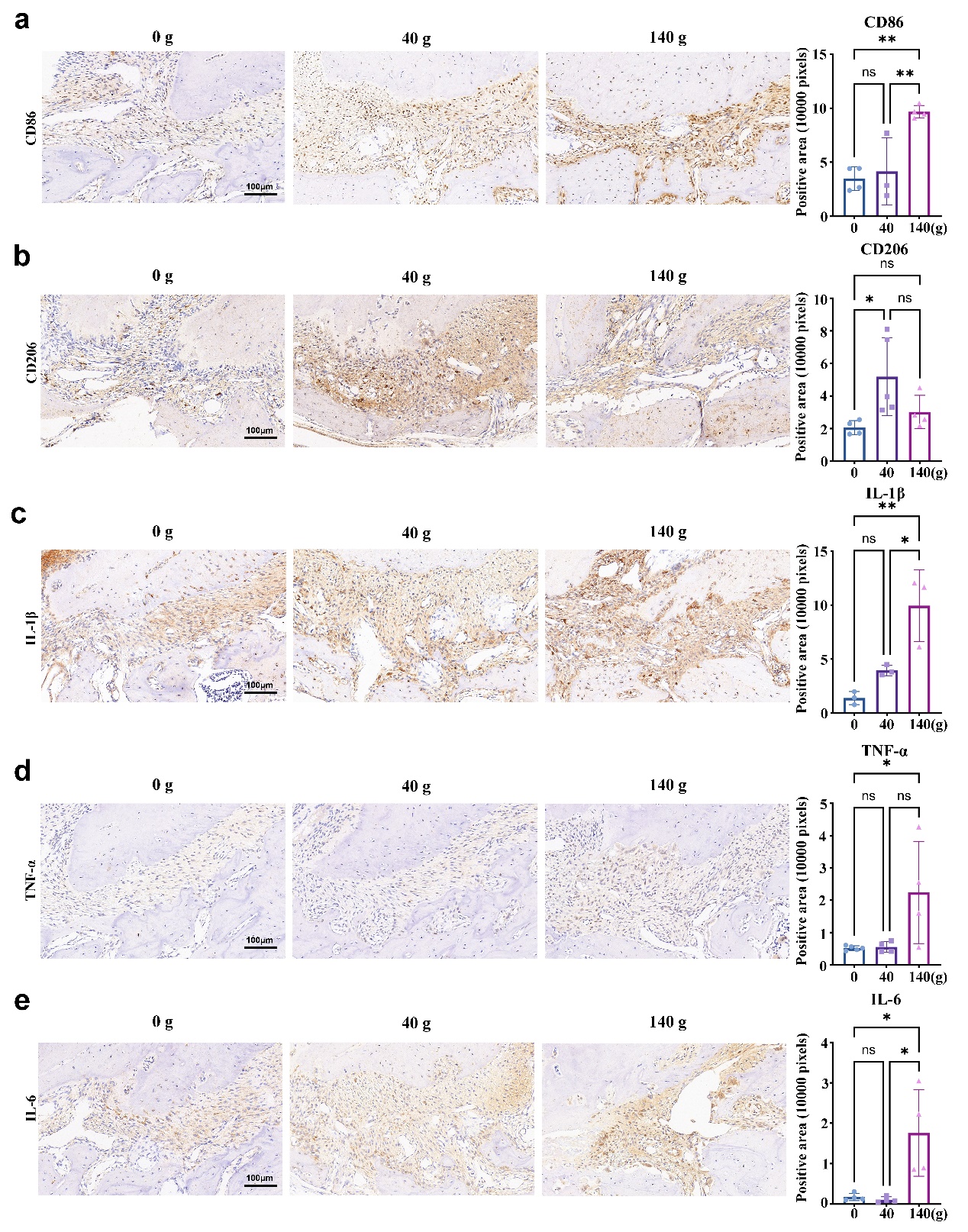
Supplementary Fig. 2 Heavy force leads to more inflammation in** **the apical distal region in the periodontal ligament of mesial roots.**

1. Immunohistochemistry staining results of groups of 0 gram, 40 grams and 140 grams show that M1-like macrophages (CD86^+^) in the apical distal region (A-D region) accumulate more in the heavy force group than that in the light force group. Mean ± SD. n=3-4. ns, not significant, ***P*<0.01 by one-way ANOVA.
2. CD206^+^ M2-like macrophages in the A-D region increase under force of 40 grams comparing to other groups. Mean ± SD. n=4-5. ns, not significant, **P*<0.05 by one-way ANOVA.

**(c-e)** Immunohistochemistry staining shows that the expression of pro-inflammatory factors IL-1β, TNF-α and IL-6 in the A-D region of periodontal tissues increases obviously in the group of 140 g. Mean ± SD. n=3-5. ns, not significant, **P*<0.05, ***P*<0.01 by one-way ANOVA.

**
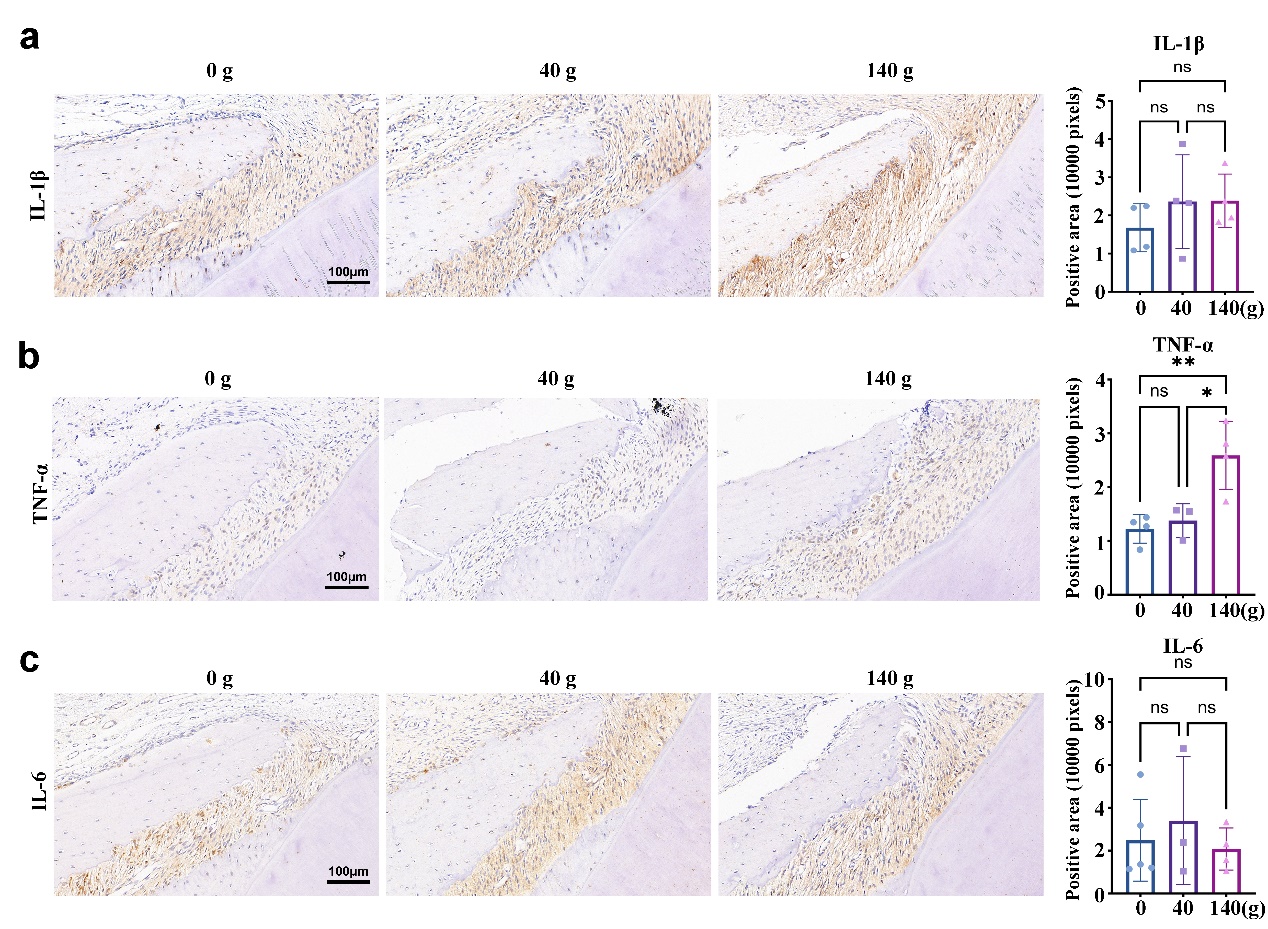
Supplementary Fig. 3 Immunohistochemistry results of inflammation indicators in the cervical mesial region of the periodontal ligament**

**(a-c)** Immunohistochemistry staining results show the expression levels of inflammatory factors IL-1β, TNF-α and IL-6 in the cervical mesial region of mesial roots. Mean ± SD. n=3-5. ns, not significant, **P*<0.05, ***P*<0.01 by one-way ANOVA.

**
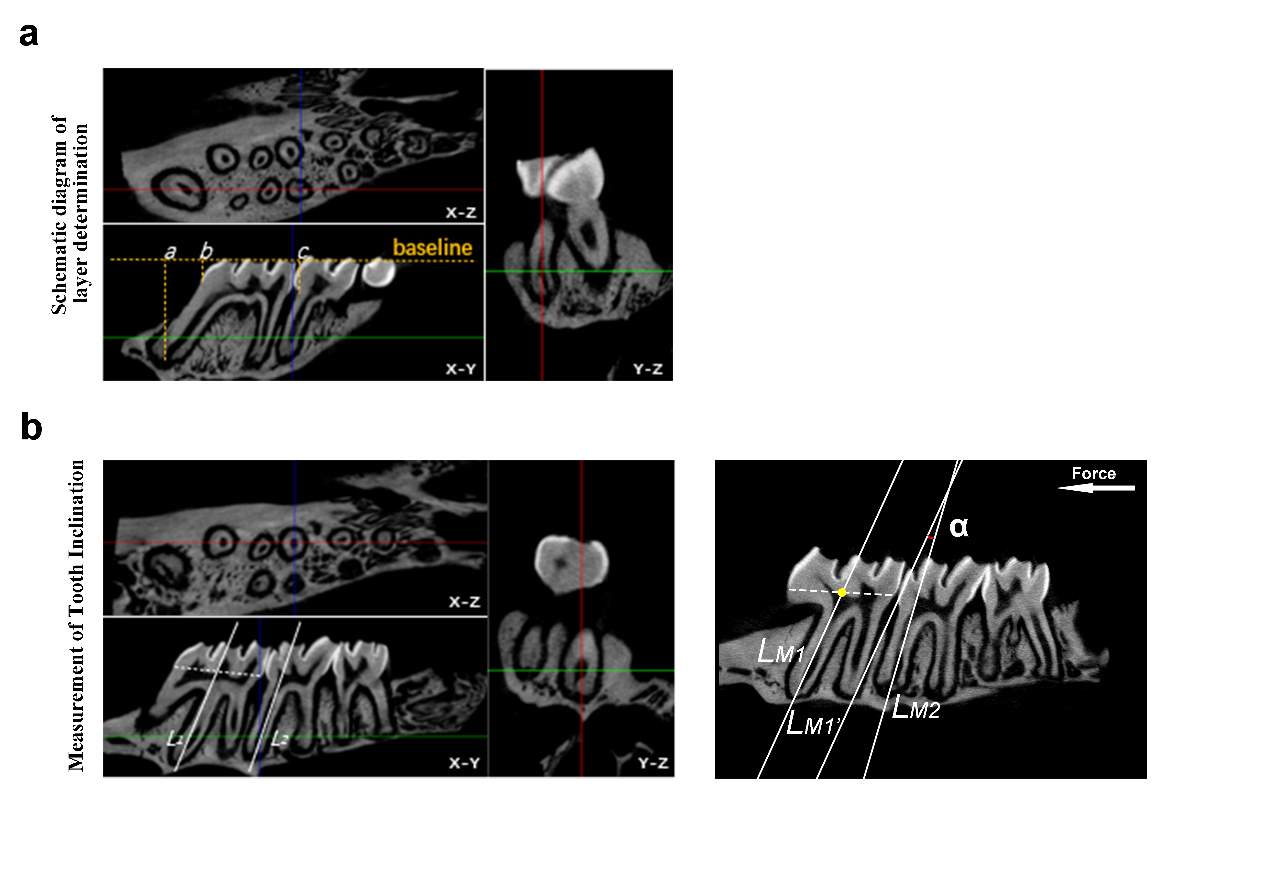
Supplementary Fig. 4 The schematic diagram of measurement methods of tooth inclination and landmarks on the occlusal plane.**

1. The image shows the layer which is used to determine the landmarks and the occlusal plane (baseline). The red line is determined by the MR of the M1 and the MBR of the M2. The intersections of perpendiculars from the apex of the MR, the mesial CEJ of M1 and M2 to the baseline were named as point a, point b and point c, representing locations of the root apex, the crown and the reference position respectively. MR mesial root, MBR mesial buccal root, M1 first molar, M2 second molar, CEJ cementoenamel junction.
2. The left image shows the layer which is used to calculate the M1 inclination and the right image shows the measurement method. The red line is determined by the MPR of the M1 and the M2. *L_M1_* was drawn from the midpoint of two CEJs to the apical foramen of the MPR, standing for the long axis of the M1. *L_M2_* was marked as the axis of MPR of M2, standing for the long axis of the M2. *L_M1_’* is parallel to *L_M1_*. α represents the tooth inclination of the M1. MR mesial root, MPR mesial palatal root, M1 first molar, M2 second molar, CEJ cementoenamel junction.


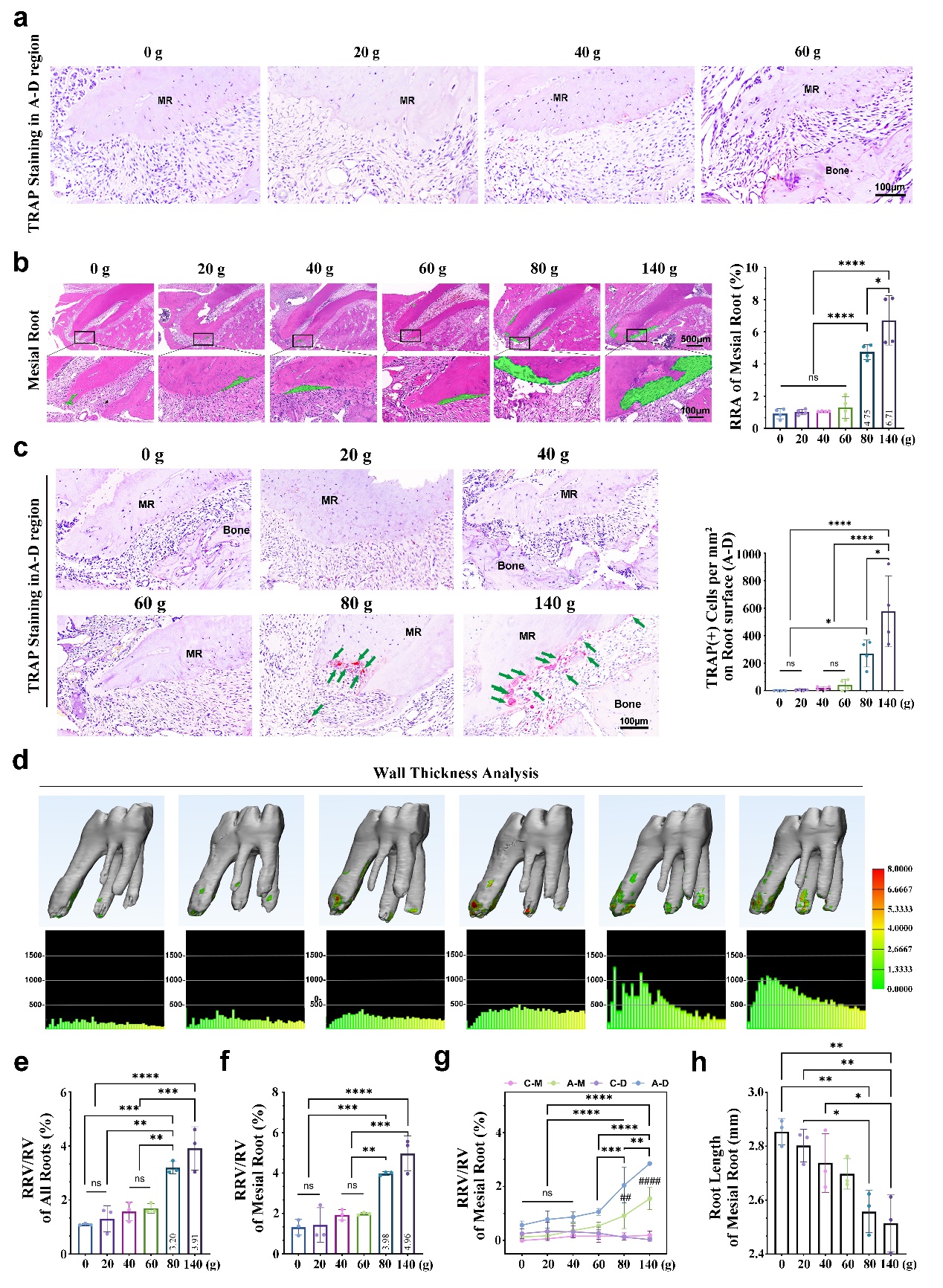
**Supplementary Fig. 5 Root resorption in the A-D region after two- or four- week force loading.**

1. TRAP staining images of 0 g, 20 g, 40 g, 60 g show the osteoclasts on the root surface (green arrows) in A-D region after two-week force loading, which indicates there is almost no root resorption.
2. HE staining shows the area of root resorption (green area) after four-week force loading. Mean ± SD. n=4. ns, not significant, **P*<0.05, *****P*<0.0001 by one-way ANOVA.
3. TRAP staining images show the osteoclasts on the root surface (green arrows) in the A-D region after four-week force loading. Mean ± SD. n=4. ns, not significant, **P*<0.05, *****P*<0.0001 by one-way ANOVA.
4. The wall thickness analysis of root resorption of the first molars after four-week force loading.
5. Statistics of root resorption volume percentages of all roots after four-week force loading. RRV root resorption volume, RV root volume. Mean ± SD. n=3. ns, not significant, ***P*<0.01, ****P*<0.001, *****P*<0.0001 by one-way ANOVA.
6. Statistics of root resorption volume percentages of mesial roots. Mean ± SD. n=3. ns, not significant, ***P*<0.01, ****P*<0.001, *****P*<0.0001 by one-way ANOVA.
7. Statistics of root resorption volume of mesial roots in different regions. Mean ± SD. n=3. * represents the statistics in the A-D region. ^#^ represents the statistics in the A-M region and comes from the comparison with the group of 0 gram. ns, not significant, ***P*<0.01, ****P*<0.001, *****P*<0.0001, ^##^*P*<0.01, ^####^*P*<0.0001 by two-way ANOVA.
8. Statistics of root length of mesial roots. Mean ± SD. n=3. ns, not significant, **P*<0.05, ***P*<0.01 by one-way ANOVA.

**
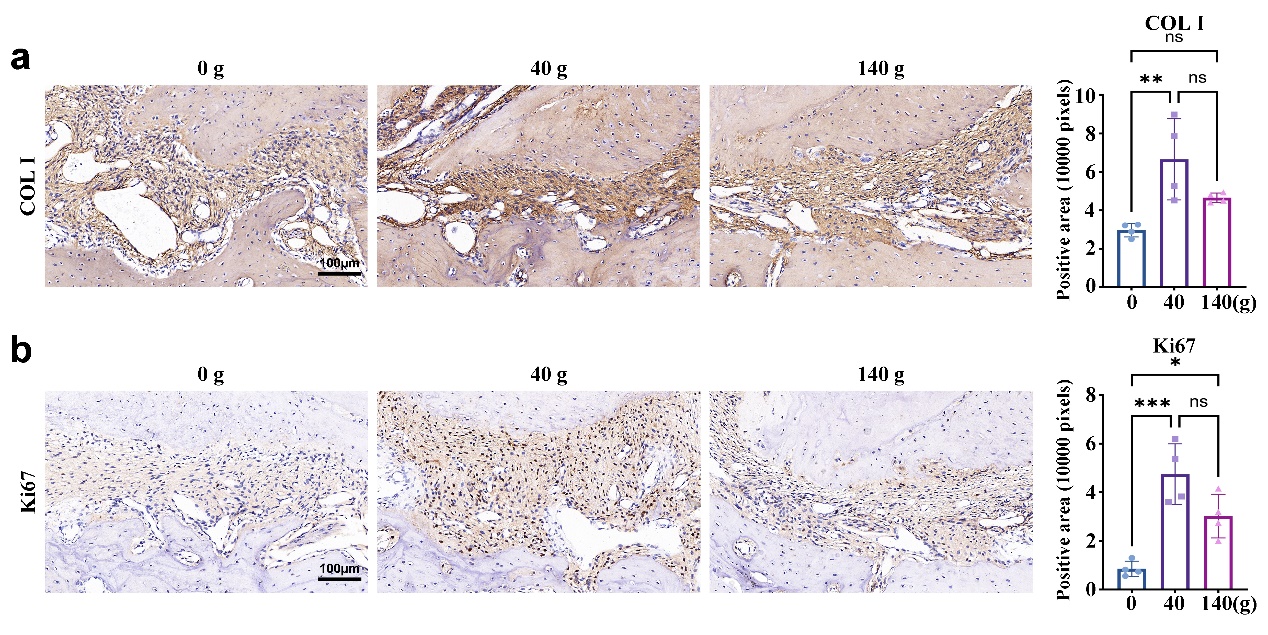
Supplementary Fig. 6 Immunohistochemistry results of indicators of collagen synthesis and cellular proliferation in the apical distal region in the periodontal ligament of mesial roots.**

1. The staining images show higher Collage I (COL I) expression level in the apical distal region in the group of 40 g than other groups. Mean ± SD. n=4. ns, not significant, ***P*<0.01 by one-way ANOVA.
2. Immunohistochemistry staining shows that the expression of Ki67 in the apical distal region of periodontal tissues increases by force and more obvious cellular proliferation is showed in the group of 40 g than 140 g. Mean ± SD. n=4. ns, not significant, **P*<0.05, ****P*<0.001 by one-way ANOVA.
